# Cerebral paragonimiasis: Clinicoradiological features and serodiagnosis using recombinant yolk ferritin

**DOI:** 10.1371/journal.pntd.0010240

**Published:** 2022-03-16

**Authors:** Jeong-Geun Kim, Chun-Seob Ahn, Insug Kang, Jong-Wook Shin, Hae-Bong Jeong, Yukifumi Nawa, Yoon Kong

**Affiliations:** 1 Department of Molecular Parasitology, Samsung Medical Center, Sungkyunkwan University School of Medicine, Suwon, Korea; 2 Department of Biochemistry and Molecular Biology, Kyung Hee University College of Medicine, Seoul, Korea; 3 Department of Internal Medicine, Chung-Ang University Hospital, College of Medicine, Chung-Ang University, Seoul, Korea; 4 Department of Neurology, Chung-Ang University Hospital, College of Medicine, Chung-Ang University, Seoul, Korea; 5 Department of Parasitology, Faculty of Medicine, University of Miyazaki, Miyazaki, Japan; University of Calgary, CANADA

## Abstract

Cerebral paragonimiasis (CP), caused by aberrant migration of *Paragonimus* worms, frequently invokes serious illness. The causal relationship between the lesion characteristics and patients’ symptoms has poorly been understood. CP serodiagnosis has not been properly evaluated to date. A total of 111 CP cases were diagnosed in our laboratory between 1982 and 2003. This study retrospectively assessed the clinical and imaging characteristics of the 105 patients along with the evaluation of diagnostic potentials of recombinant *P*. *westermani* yolk ferritin (rPwYF) by enzyme-linked immunosorbent assay (ELISA) employing patients’ sera and cerebrospinal fluids (CSFs). We analyzed 60 male and 45 female patients; 50 early-stage patients with non-calcified enhancing nodule(s) (median age, 38 years; interquartile range [IQR], 24.75–52; median symptom duration, 0.75 years; IQR, 0.2–2) and 54 chronic cases having calcified lesion(s) (median age, 33 years; IQR, 25–41; median symptom duration, 10 years; IQR, 5–20). One patient showed a normal neuroimage. The patients were largely diagnosed in their 30s. The parietal lobe was most commonly affected, followed by occipital, frontal, and temporal lobes. Twenty-six patients had lesions encompassing ≥ two lobes. The patients complained mainly of seizures, headaches, hemiparesis, and focal neurologic deficits (*P* < 0.001). Seizures and visual defects were predominant in patients with calcified lesion(s) (*P* < 0.001). The diagnostic sensitivity and specificity of rPwYF against serum/CSF were 100%/97% and 97.2%/92.5%, respectively. The specific IgG antibody levels against rPwYF in sera and CSFs showed a positive correlation (r = 0.59). The clinical manifestations of the early-stage patients might be associated with cortical lesions or meningeal irritation, while those in the chronic stage were caused by conglomerated space-occupying lesions. rPwYF would be useful for the serodiagnosis of both early and chronic CP cases.

## Author summary

Paragonimiasis, caused by the lung fluke *Paragonimus* spp., is a global foodborne zoonosis of great public health concern. Paragonimiasis is primarily a pleuropulmonary disease. However, the worms sometimes migrate aberrantly to other parts of the body, thereby causing the granulomatous lesion(s). The most serious condition elicited by ectopic migration is cerebral paragonimiasis. This study retrospectively analyzed the clinical and imaging features of the largest number of 105 cases diagnosed between 1982 and 2003 in our laboratory. We also evaluated diagnostic feasibility of recombinant *P*. *westermani* yolk ferritin by ELISA. The patients were largely diagnosed in their 30s with the chief complaints of seizures, headaches, hemiparesis, and focal neurologic deficits. Seizures and visual defect were significant in the chronic stage patients having calcified lesion(s). The diagnostic sensitivity and specificity of recombinant *P*. *westermani* yolk ferritin by ELISA against patients’ sera or CSFs were 100% or 97%, and 97.2% or 92.5%, respectively. The diverse neurological manifestations of patients might be related to the nature and/or location of the lesions. *P*. *westermani* yolk ferritin is a promising antigen for serodiagnosis of both early-stage patients with non-calcified lesion(s) and chronic calcified cerebral paragonimiasis cases.

## Introduction

Paragonimiasis is a major foodborne helminthiasis caused by infection with the genus *Paragonimus*. More than 50 species are widely distributed in different regions of the world, of which at least seven major species complexes triggered human infection [1]. The consumption of raw or undercooked freshwater crabs or crayfish harboring metacercaria(e) is the main source of human infections. Eating raw wild boar, rodent, or venison is also associated with the transmission of human paragonimiasis [2]. Approximately 20 million people are infected globally and another 300 million are at risk of infection annually [3].

Paragonimiasis is primarily a pleuropulmonary disease, which is manifested by variable respiratory symptoms depending upon the stage of infection [4,5]. However, the worms sometimes migrate aberrantly to other parts of the body, causing the granulomatous lesion(s) in the affected sites. The most serious condition elicited by ectopic migration is cerebral paragonimiasis (CP), which presents with diverse neurological symptoms and even death [6,7].

The diagnosis of CP is not a straightforward task, as eggs are not detected in the patients, except for patients who have coinfections in the lungs. Following approaches may provide diagnostic clues; i) history of freshwater crustacean consumption, ii) clinical manifestations, iii) imaging features, iv) laboratory results, and v) serological tests. However, clinical diagnosis is often challenging as non-specific symptoms and signs, and medical information obtained from patients are generally of minimal diagnostic value [8,9]. More than half of paragonimiasis patients did not recall eating freshwater crustaceans, when they are diagnosed years after the onset of clinical symptoms [5].

The neuroimaging features, which consist of clusters of ring-enhancing lesions with surrounding edema (early stage) [10] or soap-bubble calcifications (chronic stage) [11–13] are compatible with CP, but most patients were initially suspected of having primary/metastatic cancer, infectious/non-infectious granuloma, cerebrovascular diseases including hemorrhage and arteriovenous malformation, or an abscess [6,12–14], probably due to the rare incidence of CP even in the endemic areas.

Early detection of CP is crucial for the proper management of patients, since early-stage patients infected with live worms could be treated with specific chemotherapeutics, which resulted in a significant reduction in disability-adjusted life years. Conversely, most chronic cases with the calcified lesion(s) require surgical intervention and are often complicated by serious sequelae [15]. Moreover, the diagnosis of CP is very difficult if the imaging findings are not pathognomonic. Under such clinical settings, serological tests may be of great value for practical and early diagnosis, but the evaluation of immuno-serological diagnostic efficacy for CP has not been properly addressed. We hypothesized that CP patients might strongly react to egg-derived proteins because numerous eggs remain for a long period to form granulomas even after the worm’s death [15,16]. Indeed, recombinant forms of *P*. *westermani* yolk ferritin (rPwYF) and egg-derived heat shock-like protein were reported to have high diagnostic applicability for pleuropulmonary paragonimiasis [17,18]. Although these proteins were not secreted, they appeared to leak into the host as the operculum ruptured and could be recognized by the host immune system.

Between 1982 and 2002, we have diagnosed 111 CP cases including three cases with spinal involvement. In this study, we analyzed the clinical and radiological characteristics of these patients and assessed the specific IgG antibody responses to rPwYF by enzyme-linked immunosorbent assay (ELISA) using serum and cerebrospinal fluid (CSF) samples of 105 patients.

## Methods

### Ethics statement

Informed consent was not required as the patients were diagnosed between 1982 to 2003. Serum samples of 42 healthy individuals were used as normal controls after receiving verbal informed consent. All serum and CSF samples were anonymized in this study. The Institutional Review Board of Samsung Medical Center, Sungkyunkwan University, Korea, approved the use of patient data and samples (protocol no. 2021-08-014).

### Study subjects and samples

From 1982 to 2003, we have diagnosed a total of 957 paragonimiasis cases in our laboratory, among which 111 patients showed neurological symptoms and signs, and imaging features compatible with CP [5]. We have assessed the clinical and radiological features of 105 patients. Since no diagnostic criteria have been established for CP, we used serological data as a reference. Clinical data of the patients which included medical history suggestive of paragonimiasis, symptoms and duration, neuroimaging features, and laboratory data were provided by the attending physicians in the forms of consultation sheets. Most of the patients and relatives were interviewed again by the corresponding author when they visited to our laboratory. The corresponding author analyzed patient’s clinical characteristics, laboratory results, and radiological findings. Six patients were excluded from the analysis due to the unavailability of demographic (three cases), clinical (one case), and neuroimaging data (two cases) (S1 Fig).

We determined anti-*Paragonimus* specific IgG antibody levels using 102 sera and 101 CSFs of 105 patients. In addition, each of 346 serum and CSF samples from patients with neurocysticercosis (149 cases), neurosparganosis (42 cases), and other neurological diseases (155 cases); i.e., tumors (27 patients), hydrocephalus (29 patients), meningitis (17 patients), calcifications (31 patients), granulomas (31 patients), and cerebrovascular diseases (20 patients) were included. These patients were diagnosed as described previously [19]. Serum samples of 42 healthy donors were used as negative controls.

### Antigens

*Paragonimus westermani* adult worm extracts (PwAWE) were prepared as described previously [20]. In brief, Intact adult *P*. *westermani* worms kept in -80°C deep freezer were thawed and homogenized with Teflon-pestle homogenizer in phosphate buffered saline (PBS) containing protease inhibitor cocktail (1 tablet per 25 ml PBS). The extracts were centrifuged at 20,000 g at 4°C for 1 h and supernatants were collected. The supernatants were used as PwAWE. A 743 bp-long coding region of PwYF (AAG17056.1) was amplified by polymerase chain reaction with gene-specific primers 5’-AGCTCATATGGCCCCGGCAAGTGTTGACTG-3’ and 5’-GAAGTCTCGAGTTAGTGAATGATGGCG G-3’, incorporating NdeI and XhoI sites (underlined sequences). The recombinant plasmid was inserted into the pET28a(+) *Escherichia coli* expression vector. The expression fidelity was confirmed by DNA sequencing. rPwYF was induced by 0.5 mM isopropyl β-D-1-thiogalactopyranoside (Sigma-Aldrich, St. Louis, MO, USA) at 37°C for 4 h. rPwYF purified by His-bind metal chelation resin (Novagen, Madison, WIS, USA) was examined by 15% reducing SDS-PAGE. Bacterial lipids were removed using Octyl-Sepharose 4 Fast Flow column (GE Healthcare, UK). The proteins were stored at -80°C.

### Enzyme-linked immunosorbent assay (ELISA)

After a checkerboard titration, 100 μl of PwAWE and rPwYF (2.5 μg/ml each) suspended in 100 mM carbonate-bicarbonate buffer (pH 9.6) were coated to the wells of a flat-bottom 96-well microplate (Greiner Bio-One, Kremsmünster Austria) overnight at 4°C. Sera diluted to 1:200 in PBS containing 0.05% Tween 20 (PBS/T) or undiluted CSFs (100 μl each) were incubated for 2 h, subsequently with 1:2,000 diluted horseradish peroxidase (HRP)-conjugated anti-human IgG antibody (100 μl; heavy- and light-chain specific, MP Biochemicals, Santa Ana, CA, USA) for 2 h. The color reaction was developed with 1% *o*-phenylenediamine (100 μl; Sigma-Aldrich) supplemented with 0.03% H_2_O_2_ for 20 min in the dark. The reaction was stopped using 2 N H_2_SO_4_ (50 μl; Sigma-Aldrich). The absorbance (abs.) was measured at 450 nm on a NEO microplate reader (Biotek, Winooski, VT, USA). All results were determined after correction with PBS/T blank.

### Statistics

We used GraphPad Prism9 software (GraphPad, San Diego, CA, USA) and SPSS statistical package (ver25, IBM, Armonk, NY, USA) for analysis of variance (ANOVA). All data are expressed as the mean ± standard deviation (SD) of at least three independent assays. A *P-*value of < 0.05 with a 2-tailed hypothesis was employed. Sensitivity, specificity, positive predictive value (PPV), negative predictive value (NPV), and 95% confidence intervals (CIs) were calculated. Receiver operating characteristic (ROC) curves were created by plotting sensitivity versus 1-specificity. The area under the ROC curve (AUC) was employed to conduct a pairwise comparison of the diagnostic performance. Cutoff values were determined by ROC analysis and defined as the positive and negative values that rendered the highest sum of sensitivity and specificity totals.

## Results

### Clinical characteristics of the study subjects

**Fig 1:**
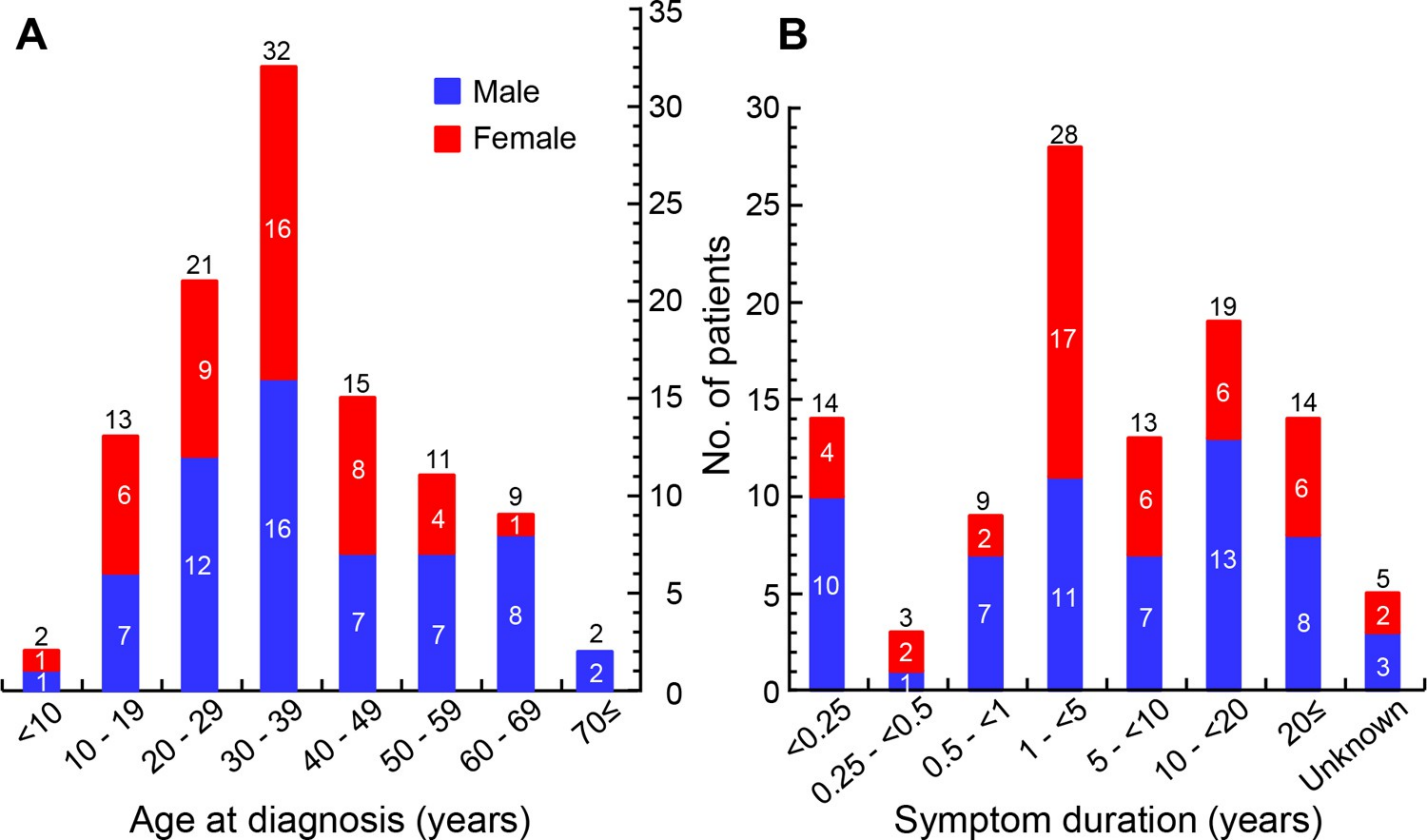
Demographic characteristics of cerebral paragonimiasis patients in this study. (A) Age distribution of patients at the time of definitive diagnosis. (B) Duration of patient symptoms until definitive diagnosis.

We analyzed 105 CP cases that comprised of 60 males and 45 females (median age, 34 years; IQR, 25–46.5; range, 5–72 years). The patients were largely diagnosed in their 30s (30.5%), followed by their 20s (20%) and 40s (14.3%) (all *P* values < 0.01) (Fig 1A). The symptom duration in the patients varied from 1 week to 35 years (median, 3.5 years; IQR, 0.625–11.75). Only 26 patients were diagnosed within 1 year of symptom onset, 28 cases were between 1–5 years of symptom onset, and 13 patients were between 5–10 years of symptom onset. The diagnosis of 33 cases was delayed for > 10 years (Fig 1B). At the initial evaluation, 37 patients (35.2%) were diagnosed with CP, whereas the remaining 68 cases (64.8%) were tentatively diagnosed with cancer, cerebrovascular diseases, hydrocephalus, or neurocysticercosis (Table 1).

**Table 1:** Initial presumptive diagnosis of cerebral paragonimiasis patients enrolled in this study.

Initial presumptive diagnosis	Lesion characteristics, n/N (%)					
	All lesions		Non-calcified lesions		Calcified lesions	
Cerebral paragonimiasis	37/105^*a*^ (35.2)		15/50 (30.0)		21/54 (38.9)	
Presumptive diagnosis	68/105 (64.8)		35/50 (70.0)		33/54 (61.1)	
Tumors^*b*^		32/68 (47.1)		15/35 (42.9)		17/33 (51.5)
Cerebrovascular diseases^*c*^		14/68 (20.6)		10/35 (28.6)		4/33 (12.1)
Hydrocephalus		8/68 (11.8)		4/35 (11.4)		4/33 (12.1)
Neurocysticercosis		8/68 (11.8)		3/35 (8.6)		5/33 (15.2)
Cerebromalacia		3/68 (4.4)		1/35 (2.9)		2/33 (6.1)
Granuloma/cyst(s)^d^		2/68 (2.9)		1/35 (2.9)		1/33 (3.0)
Tuberculosis		1/68 (1.5)		1/35 (2.9)		0/33 (0.0)

^*a*^The total number included non-calcified lesions (*n* = 50), calcified lesions (*n* = 54), and normal finding (*n* = 1).

^*b*^Tumors included primary and metastatic cancers.

^*c*^Cerebrovascular diseases included hemorrhage, ischemia, acute and old infarcts, and arteriovenous malformations.

^*d*^Cysts included cavitary lesions.

**Fig 2:**
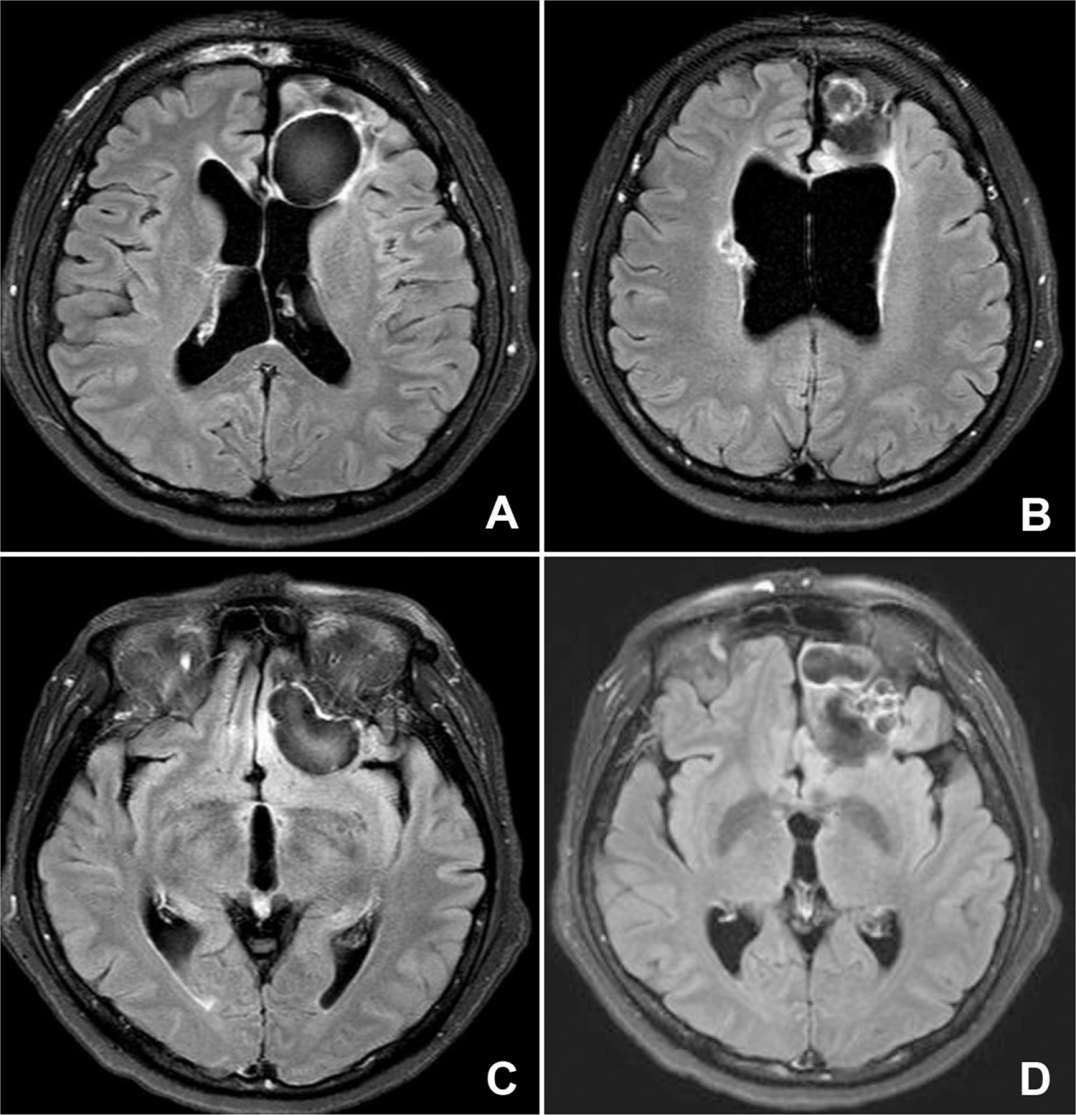
Fluid attenuated inversion recovery (FLAIR) sequence of brain magnetic resonance image of a 68-year-old female patient who suffered from dysarthria and right-sided weakness for 9 years. The laboratory results including eosinophil counts were within the normal range. The patient could not recall the consumption of freshwater crustaceans. The anti-*Paragonimus* specific IgG antibody levels against adult worm extracts were 0.31 (serum) and 0.26 (CSF), respectively. (A and B) FLAIR sequence shows cerebromalacia and ventriculomegaly with a round calcified mass on the left frontal lobe. (C and D) Several well-defined calcified masses in the left inferior frontal lobe are seen.

Table 2 summarized clinical features along with lesion localities. The patients mainly complained of seizures (54.3%), headache (51.4%), and hemiparesis (20%) (all *P* values < 0.001). The parietal (26.3%), occipital (25.5%), frontal (19%), and temporal lobes (14.6%) were commonly affected (all *P* values < 0.001). Lesions involved ≥ two lobes were found in 26 cases (24.8%, *P* < 0.001). Hydrocephalus combined with an intraventricular cavity, or calcified nodules (Fig 2) was recognized in 11 patients (10.5%, *P* < 0.01) (median symptom duration, 8 years; IQR, 2.25–10). Lesions in the cerebellum, spinal cord, and thalamus were found in 3, 3, and 1 cases, respectively (Table 2). Eggs were detected in 15 patients either by surgery or biopsy (13 patients), or sputum examination (two cases that were coinfected in the lungs). Eating freshwater crustaceans was recalled by 41 patients (39%, *P* < 0.05). Coinfections in the lungs and subcutaneous tissues were detected in 11 and 2 cases, respectively. One patient had multiple infections in the frontal lobe, thoracic spinal cord, and lungs.

**Table 2:** Relationship between lesion locations and clinical manifestations of cerebral paragonimiasis patients.

Lesion location, n/N (%)		Symptoms, n/N (%)						*P-*values
		Headache	Seizure^*a*^	Hemiparesis^*b*^	FND^*c*^	Visual defects^*d*^	Mental impairment^*e*^	
Parietal lobe	36/137^*f*^ (26.3)	15/36 (41.7)	21/36 (58.3)	8/36 (22.2)	6/36 (16.7)	4/36 (11.1)	3/36 (8.3)	< 0.001
Occipital lobe	35/137 (25.5)	20/35 (57.1)	20/35 (57.1)	5/35 (14.3)	3/35 (8.6)	7/35 (20.0)	1/35 (2.9)	< 0.001
Frontal lobe	26/137 (19.0)	12/26 (46.2)	16/26 (61.5)	6/26 (23.1)	2/26 (7.7)	0/26 (0.0)	2/26 (7.7)	< 0.001
Temporal lobe	20/137 (14.6)	9/20 (45.0)	13/20 (65.0)	3/20 (15.0)	6/20 (30.0)	3/20 (15.0)	1/20 (5.0)	< 0.001
Ventricle	11/137 (8.0)	7/11 (63.6)	2/11 (18.2)	4/11 (36.4)	5/11 (45.5)	1/11 (9.1)	0/11 (0.0)	0.0058
Cerebellum	3/137 (2.2)	1/3 (33.3)	1/3 (33.3)	1/3 (33.3)	0/3 (0.0)	0/3 (0.0)	0/3 (0.0)	-
Spinal cord	3/137 (2.2)	0/3 (0.0)	1/3 (33.3)	2/3 (66.7)	0/3 (0.0)	0/3 (0.0)	0/3 (0.0)	-
Brain stem	1/137 (0.7)	1/1 (100.0)	0/1 (0.0)	0/1 (0.0)	1/1 (100.0)	0/1 (0.0)	0/1 (0.0)	-
Thalamus	1/137 (0.7)	0/1 (0.0)	0/1 (0.0)	1/1 (100.0)	1/1 (100.0)	0/1 (0.0)	0/1 (0.0)	-
Normal	1/137 (0.7)	1/1 (100.0)	1/1 (100.0)	0/1 (0.0)	0/1 (0.0)	0/1 (0.0)	0/1 (0.0)	-

^*a*^Seizure included partial and generalized seizures.

^*b*^Hemiparesis included hemiplegia and paraplegia.

^*c*^Focal neurologic deficits (FND) included sensory/motor impairment, gait disturbance, urinary difficulty, proprioception impairment, dysphasia, dyslexia, dyscalculia, and agnosia.

^*d*^Visual defects included blurred vision, diplopia, anorthopia, hemianopsia, quadrantanopia, and scotomata.

^*e*^Mental impairment included mental retardation, intellectual disability, cognition difficulty, apathy, and memory impairment.

^*f*^Total numbers exceed 105 due to extensive conglomerated lesions spanning the parietooccipital (*n* = 8), parietotemporal (*n* = 7), temporoocipital (*n* = 6), frontoparietal (*n* = 3), frontotemporal (*n* = 1), and temporoparietooccipital lobes (*n* = 1).

We categorized 105 patients into two groups of early and chronic cases according to their neuroimages (S1 Fig). Fifty early cases had a single or multiple hypodense mass lesion(s) with/without enhancement and 54 chronic cases had a single or conglomerated calcified lesion(s). One patient demonstrated no obvious lesion on imaging studies (for details, refer to the following paragraph). The symptom duration (median, 0.75 years [IQR, 0.2–2] versus 10 years [IQR, 5–20]) and clinical manifestations were significantly different between the groups (all *P* values < 0.001). Seizures and visual defect constituted the majority of the symptoms in the patients with calcified lesions (*P* < 0.001) (S1 Table). Gender differences were not observed between the groups (*P* > 0.05).

Interestingly, a 36-year-old female patient with a generalized seizure, headache, focal neurologic deficits, and visual field defect for the past 2 months showed no visible lesions on imaging studies. Laboratory results including eosinophil counts were within the normal ranges. She had consumed pickled freshwater crab approximately a month before symptom onset. She was referred to our laboratory to rule out the presence of parasitic meningitis. The anti-*Paragonimus* specific IgG antibody levels in the serum and CSF were 0.41 and 0.46, respectively, against PwAWE. She was administered praziquantel (75 mg/kg per day for 2 weeks), together with medications to relieve the neurological symptoms. Her serum and CSF antibody levels spiked to 0.8 and 0.71 during 1-month post-treatment, thereafter the values gradually decreased to 0.36 and 0.29, respectively, at the 6-month follow-up. Her neurological symptoms resolved slowly over 8 months.

### Comparison of diagnostic potential of PwAWE and rPwYF

**Fig 3:**
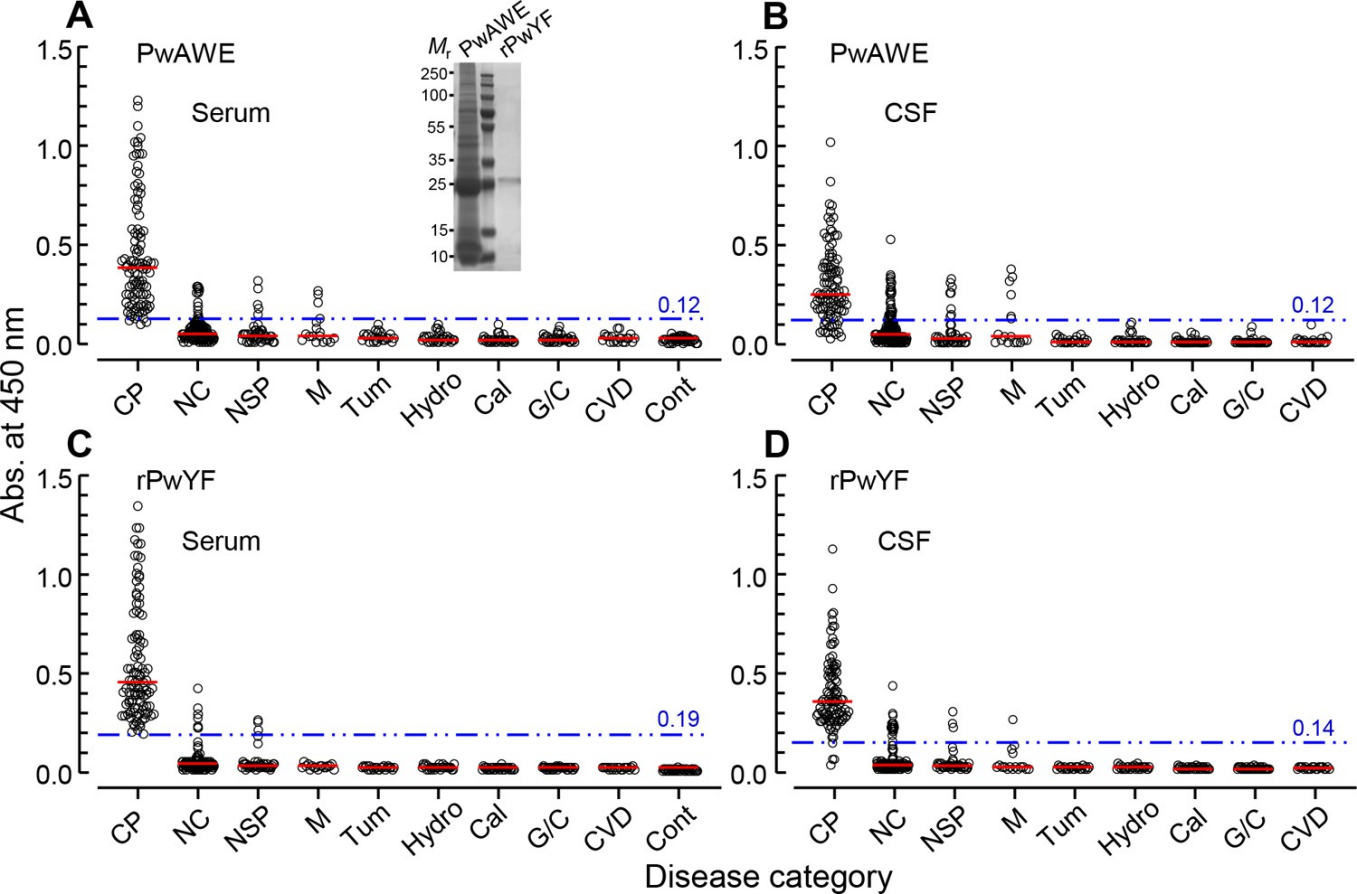
Comparative evaluation of the diagnostic performance of *P*. *westermani* adult worm extracts (PwAWE) and recombinant *P*. *westermani* yolk ferritin (rPwYF). Specific IgG antibody levels in the serum and CSF samples of patients with various neurological diseases and those in the sera of healthy controls against PwAWE (A and B) and rPwYF (C and D). The *y*-axis shows the absorbance values of the tested sera and CSFs. The *x*-axis depicts the following groups: CP, cerebral paragonimiasis (*n* = 102 [serum] and *n* = 101 [CSF]); NC, neurocysticercosis (*n* = 149); NSP, neurosparganosis (*n* = 42); M, meningitis (*n* = 17); Tum, tumor (*n* = 27); Hydro, hydrocephalus (*n* = 29); Cal, calcification (*n* = 31); G/C, granuloma/cystic mass (*n* = 31); CVD, cerebrovascular diseases (*n* = 20); Cont, healthy controls (*n* = 42). The cutoff values determined by receiver operating characteristic analysis shown in Fig 4 are marked by double-dotted blue lines. The red bars indicate the mean absorbance in each group. Inset in panel A shows the protein profiles of PwAWE and rPwYF separated by 15% reducing SDS-PAGE. *M*_r_, molecular weight in kDa.

**Fig 4:**
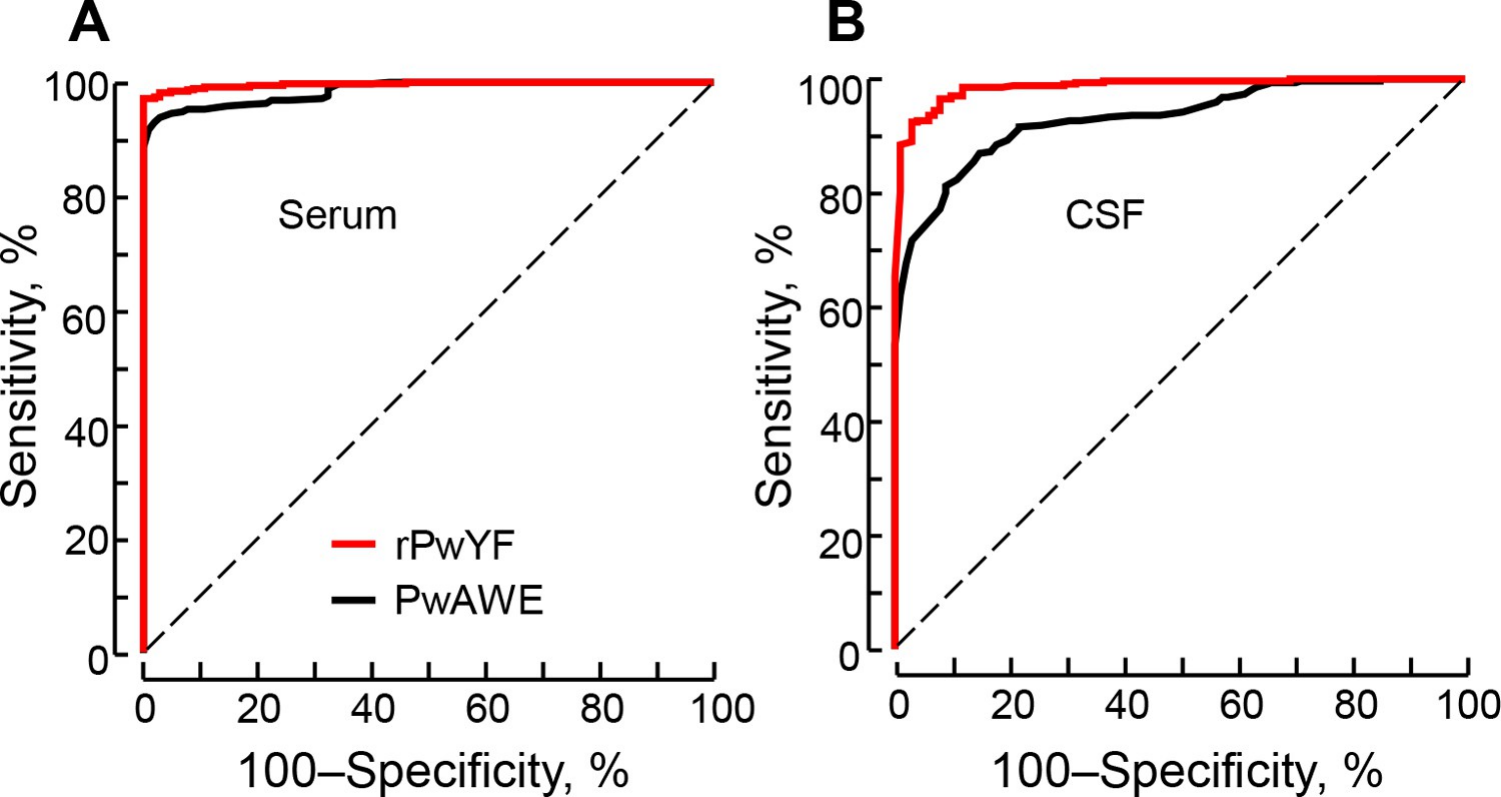
Analysis of receiver operating characteristic (ROC) curves. The ROC curves demonstrate the plots of sensitivity versus 1-specificity for serum (A) and CSF samples (B) against adult worm extract (PwAWE) (black line) and recombinant *P*. *westermani* yolk ferritin (rPwYF) (red line), respectively. The diagonal reference line is also shown. ROC curves were used to determine the cutoff values and area under the curve (AUC) each for PwAWE and rPwYF in serum (*n* = 102) and CSF (*n* = 101) samples by ELISA in cerebral paragonimiasis cases.

We comparatively evaluated the specific IgG antibody levels against two different antigen preparations, PwAWE and rPwYF (Fig 3). The serum and CSF samples from CP patients exhibited fairly high antibody responses against both the antigens. Table 3 demonstrated the diagnostic performance of PwAWE and rPwYF. The optimal cutoff abs. of 0.12 in both serum and CSF samples for PwAWE distinguished the positive and negative reactions with 95% CIs of 0.98–0.99 and 0.91–0.95, respectively. ELISA values for rPwYF of 0.19 (serum) and 0.14 (CSF) differentiated positive and negative reactions with 95% CIs of 0.99–1.00 and 0.98–1.00, respectively. The AUCs for serum and CSF against PwAWE were 0.986 ± 0.004 and 0.931 ± 0.012, and those against rPwYF were 0.995 ± 0.002 and 0.987 ± 0.004, respectively. The ROC curve analysis indicated that rPwYF was a more reliable antigen for the CP serodiagnosis (Fig 4). The relationship between specific IgG antibody levels against PwAWE (r = 0.58) and rPwYF (r = 0.59) in sera and CSFs revealed a positive correlation (S2A and S2B Fig). The analysis of specific antibody levels between serum (r = 0.99) and CSF (r = 0.97) against PwAWE and rPwYF revealed high correlation (S2C and S2D Fig).

**Table 3:** Evaluation of the biomarker potential of different antigenic preparations for the serodiagnosis of cerebral paragonimiasis.

	Adult worm extracts				Recombinant yolk ferritin			
	Positive, *n/N*				Positive, *n/N*			
	Serum	CSF	Both^*a*^	Total^*b*^	Serum	CSF	Both^*a*^	Total^*b*^
CP	100/102	86/101	82/98	104/105	102/102	98/101	95/98	105/105
Overall	27/388	45/346	53/346	19/388	11/388	26/346	29/346	8/388
Neurocysticercosis	18/149	31/149	38/149	11/149	8/149	22/149	24/149	6/149
Neurosparganosis	5/42	8/42	9/42	4/42	3/42	3/42	4/42	2/42
Meningitis^*c*^	4/17	6/17	6/17	4/17	0/17	1/17	1/17	0/17
Others^*d*^	0/138	0/138	0/138	0/138	0/138	0/138	0/138	0/138
Controls	0/42	NA	NA	0/42	0/42	NA	NA	0/42
Sensitivity, %	98.0	85.2	83.7	99.0	100.0	97.0	96.9	100.0
Specificity, %	93.0	87.0	84.7	95.1	97.2	92.5	91.6	97.9
PLR	14.09	6.55	5.46	20.23	35.30	12.91	11.57	48.50
NLR	0.02	0.17	0.19	0.01	0.00	0.03	0.03	0.00
PPV, %	78.7	65.7	60.7	84.6	90.3	79.0	76.6	92.9
NPV, %	99.5	95.3	94.8	99.7	100.0	99.1	99.1	100.0
AUC ± SE	0.986 ± 0.004	0.931 ± 0.012	NA	NA	0.995 ± 0.002	0.987 ± 0.004	NA	NA
95% CI	0.98, 0.99	0.91, 0.95	NA	NA	0.99, 1.00	0.98, 1.00	NA	NA
Cutoff^*e*^	0.12	0.12	NA	NA	0.19	0.14	NA	NA

AUC, area under the curve; CP, cerebral paragonimiasis; NA, not available; NLR, negative likelihood ratio; NPV, negative predictive value; SE, standard error; PLR, positive likelihood ratio; PPV, positive predictive value.

^*a*^Number of patients whose sera and CSFs showed positive reactions.

^*b*^Number of patients whose sera or CSFs showed positive reaction.

^*c*^Meningitis included infections due to bacteria, fungus, and tuberculosis.

^*d*^Others included tumors, hydrocephalus, calcifications, granulomas, and cerebrovascular diseases.

^*e*^Cutoff values were determined by the analysis of ROC curves.

### rPwYF showed highly consistent diagnostic performance

Both PwAWE and rPwYF exhibited appreciable antibody-capturing activity in the sera and CSFs obtained from CP patients, but rPwYF showed less cross-reactivity with other neurological patients compared to PwAWE (Fig 3). As shown in Table 4, specific antibody levels against rPwYF were also high in both the serum and CSF (*P* < 0.05). The diagnostic sensitivity of the serum and CSF tests against rPwYF was 100% and 97%, and that of PwAWE was 98% and 85.2%, respectively. PwAWE showed much lower sensitivity to CSFs than to sera. This pattern was more pronounced in patients with calcified lesions. rPwYF demonstrated 100% (serum) and 94.4% (CSF) positivity, while PwAWE showed 98% (serum) and 79.6% (CSF) positivity, respectively, in diagnosing calcified patients. The diagnostic specificity of rPwYF and PwAWE was 97.9% (380/388 cases) and 95.1% (369/388 cases), respectively. PwAWE exhibited more frequent cross-reactions with CSFs obtained from patients with neurocysticercosis, neurosparganosis, and meningitis than rPwYF (Table 3). Of the 98 patients whose serum and CSF were tested simultaneously, 95 patients (96.9%) demonstrated positive responses in both the samples against rPwYF, whereas 82 cases (83.7%) showed positive responses against PwAWE. The PPV and NPV of rPwYF were 92.9% and 100%, while those of PwAWE were 84.6% and 99.7%, respectively (Tables 3 and 4).

**Table 4:** Comparison of the diagnostic efficacy of serological biomarkers for cerebral paragonimiasis.

Antigens	Lesion characteristic	Positive, *n/N*				Sensitivity, %				Specific antibody levels^*a*^	
		Serum	CSF	Both^*b*^	Total^*c*^	Serum	CSF	Both^*b*^	Total^*c*^	Serum	CSF
Adult extracts	Overall	100/102	86/101	82/98	104/105	98.0	85.2	83.7	99.0	0.45 ± 0.28	0.30 ± 0.19
	Non-calcified lesions	49/50	42/46	41/46	50/50	98.0	91.3	89.1	100.0	0.60 ± 0.29	0.36 ± 0.20
	Calcified lesions	50/51	43/54	40/51	53/54	98.0	79.6	78.4	98.1	0.31 ± 0.18	0.25 ± 0.16
	Normal	1/1	1/1	1/1	1/1	100.0	100.0	100.0	100.0	0.41	0.46
Recombinant yolk ferritin	Overall	102/102	98/101	95/98	105/105	100.0	97.0	96.9	100.0	0.55 ± 0.29	0.39 ± 0.18
	Non-calcified lesions	50/50	46/46	46/46	50/50	100.0	100.0	100.0	100.0	0.70 ± 0.30	0.44 ± 0.19
	Calcified lesions	51/51	51/54	48/51	54/54	100.0	94.4	94.1	100.0	0.40 ± 0.18	0.34 ± 0.19
	Normal	1/1	1/1	1/1	1/1	100.0	100.0	100.0	100.0	0.39	0.51

^*a*^Specific antibody levels are presented as mean ± SD.

^*b*^Number of patients whose serum and CSF showed positive reactions.

^*c*^Number of patients whose serum or CSF showed positive reaction.

## Discussion

This retrospective study analyzed the largest number of CP cases ever studied and investigated the clinical manifestations and imaging features along with the nature and location of the lesions, thus to seek possible causal relationships between the lesion characteristics and patients’ symptoms. We also assessed the serodiagnostic reliability of rPwYF using serum and CSF samples from these patients and showed that rPwYF would be a promising biomarker for serodiagnosis of CP patients.

CP accounted for 11.6% of the paragonimiasis cases (111/957 cases) (S1 Fig), which was consistent with previous reports. Population-based studies reported that CP accounted for 0.2–27% of the paragonimiasis cases in Korea in the 1950s and 1960s [11,21]. Our study indicated that the incidence of CP was the highest among those in their 30s without any gender difference (Fig 1A). Given the symptom duration, these patients might have been infected in their 20s by eating pickled freshwater crabs.

*Paragonimus* frequently invades the parieto-occipital area of the brain [11,13,15]. However, in this study, lesions involving the frontal lobe and conglomerated lesions encompassing ≥ two lobes were also significant (*P* < 0.01), the data being consistent with previous reports [22–25]. How lung flukes migrate to the brain is not completely understood, but the worms may invade the brain by migrating through the loose muscular gaps between the vertebral column, or along with soft tissues surrounding the carotid artery and small vessels [15,22]. Once having invaded the cerebral hemisphere, the worms continue to wander dynamically, leaving a migration track [23]. The active motility of the worm and tissue-hydrolyzing proteolytic enzymes secreted by the worm [26,27] are the main driving forces for long-distance migration.

We classified CP patients into two groups based on the neuroimaging features, those having non-calcified lesion(s) and those with the calcified lesion(s), which might be closely related to the infection duration. The chief complaints of CP patients included headaches, seizures, hemiparesis, and focal neurological deficits, which appeared to be dependent upon the nature or locality of the lesions (Table 2). Headache might be caused by lobar and cortical lesions associated with inflammatory responses and meningeal irritation [28]. Cortical lesions with/without calcification in the occipital, parietal, frontal, and temporal lobes have been reported to invoke various types of seizures [29]. Huge eggshell-like calcifications developed during the long-term healing process from the worm’s mechanical and biochemical damages might act as an epileptogenic focus, thereby inducing various types of seizures in patients with calcified lesions. Injury to the motor nerve in the cerebral hemisphere and motor tracts in the spinal cord might lead to hemiparesis. Conglomerated calcified lesions spanning the visual cortex and visual tract might provoke visual defects [30]. Contrary to previous studies [11,12,24], mental impairment could not be observed in a large number of patients in this study, probably due to fewer heavy infections.

Our patients with non-calcified lesions might represent early-stage meningitis/meningoencephalitis form and those with calcified lesions might be compatible with chronic extensive space-occupying form as previously proposed [15,25]. In addition, another form of CP combined with hydrocephalus was found in a considerable number of patients (24.8%, *P* < 0.001). Since hydrocephalus causes persistent brain damage and even death due to herniation by increased intracranial pressure [31], physicians should be cautious when managing patients with such symptoms.

Many CP patients were misdiagnosed at the initial evaluation or diagnosed belatedly after symptoms persisted for quite a long period (Table 1 and Fig 1B). The neuroimaging features of CP are often confused for cancers, cerebrovascular diseases, or other parasitic infections. Paragonimiasis, which had been prevalent in Korea, has significantly declined since the 1970s [5,32]. The low incidence of CP has resulted in a higher rate of misdiagnosis and/or delayed diagnosis [33]. Even among the developed countries where paragonimiasis is still endemic, attentions should be paid to the differential diagnosis of CP from other organic brain diseases.

The criteria for serological diagnosis of pleuropulmonary paragonimiasis have well been established [4,5,15,20], while those for CP have poorly been evaluated due to the difficulty in obtaining a significant number of sera and/or CSFs. Moreover, a molecularly well-defined antigen is essential to undertake specific serodiagnosis. This study assessed the serological feasibility of rPwYF. We selected rPwYF as an alternative antigen for three specific reasons. First, most helminths that invade the central nervous system (CNS) in Korea are larval forms, like *Taenia solium* metacestode and sparganum. *P*. *westermani* exceptionally grows into an egg-laying adult in the CNS. Consequently, cross-reactivity against yolk ferritin might be decreased as other larval helminths invading the CNS do not express yolk ferritin [34]. Since there is a positive correlation between serum and CSF levels of specific IgG antibodies against rPwYF (r = 0.59, S2A and S2B Fig), we surmise that specific IgG antibody levels in CSF may reflect those of serum. Second, *P*. *westermani* eggs remain unresolved for quite a long period after the fluke dies [15,16], thereby allowing for easy detection of specific antibodies against egg-derived yolk ferritin. Last, the low sequence homology of PwYF with other parasitic homologs [17] may favor high specificity without compromising sensitivity.

In this study, rPwYF demonstrated a reproducible and reliable potential for serodiagnosis of CP cases (Tables 3 and 4). The cutoff values employed in this study were lower than those applied for pleuropulmonary paragonimiasis [5,17,20] but resulted in a good performance. rPwYF was effective not only for the serodiagnosis of early-stage non-calcified patients, but also for diagnosis of calcified CP (Table 4). Since calcified CP lesions continuously trigger various neurological symptoms, differential diagnosis of chronic calcified CP cases from other brain pathologies appears to be also important. When we tested both samples of serum and CSF simultaneously, some cases gave positive response in only one sample. The positive correlation of specific antibody levels between serum and CSF strongly suggests that a positive response in one sample might be applicable (S2A and S2B Fig). The use of serum sample would be appreciable for CP serodiagnosis. A few serum and CSF samples from neurocysticercosis, neurosparganosis, and tuberculosis meningitis patients showed non-specific positive reactions possibly due to severe inflammatory conditions during the disease progression.

This study includes several shortcomings. We determined anti-rPwYF specific antibody levels of total IgG, but not those of IgG4. It has been reported that the detection of specific IgG4 antibodies is sensitive in the serological diagnosis of helminthic infections [35]. However, this study evaluated total IgG antibody levels because we have previously demonstrated that detection of total IgG offered a diagnostic value equivalent to that of IgG4 [36]. In addition, since we had detected total IgG antibody levels while diagnosing CP patients enrolled in this study, we determined the diagnostic potential of rPwYF under similar conditions. We did not examine serological responsiveness to rPwYF in patients infected with *P*. *skrjabini*. *P*. *skrjabini* does not mature into an adult in the human body, but the juvenile can migrate to the CNS and cause CP [9,22,23]. Whether specific antibodies against *P*. *skrjabini* yolk ferritin are induced and whether these antibodies are detectable by rPwYF require further investigation. We did not analyze laboratory data because many patients showed no pathological findings, including eosinophilia (4 cases had eosinophilia with a count > 7%). We could not trace the treatment outcomes due to the lack of follow-up surveillance in most cases.

In conclusion, CP is sporadically detected in Korea. The symptoms of CP patients might be dependent on the location and/or nature of the lesions. The detection of specific IgG antibodies in the serum or CSF against rPwYF would be highly beneficial for the serodiagnosis of CP.

## Supporting information

S1 Table Comparison of clinical manifestations according to lesion characteristics. (TIF)

S1 Fig Study profile. CP = Cerebral paragonimiasis.
(TIF)

S2 Fig Correlation of specific IgG antibody levels in serum and CSF samples of cerebral paragonimiasis patients against *P*. *westermani* adult worm extracts (PwAWE) and recombinant *P*. *westermani* yolk ferritin (rPwYF). (A and B) Relationship between specific IgG antibody levels against PwAWE and rPwYF in serum and CSF samples from patients with cerebral paragonimiasis. (C and D) Relationship of specific IgG antibody levels between serum and CSF against PwAWE and rPwYF. The horizontal and vertical double dotted blue lines indicate positive criteria.
(TIF)
