# Supplementary figures and images for: Cerebral paragonimiasis: Clinicoradiological features and serodiagnosis using recombinant yolk ferritin
Source: PLoS Negl Trop Dis. 2022 Mar 16;16(3):e0010240. doi: 10.1371/journal.pntd.0010240 (PMC8959177; doi:10.1371/journal.pntd.0010240)

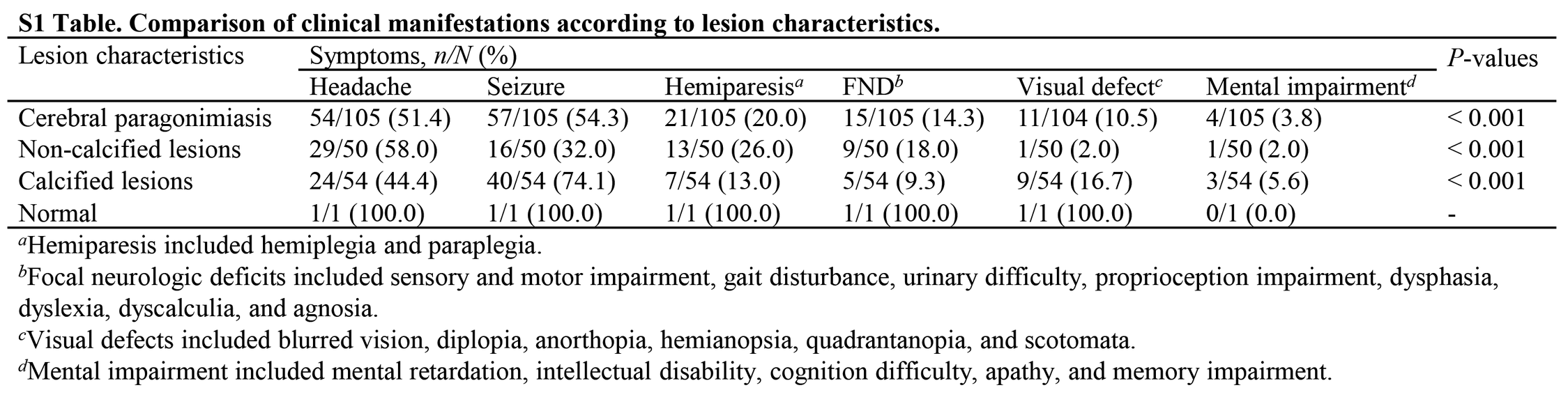

Supplement: S1 Table — (TIF) [file pntd.0010240.s001.tif]

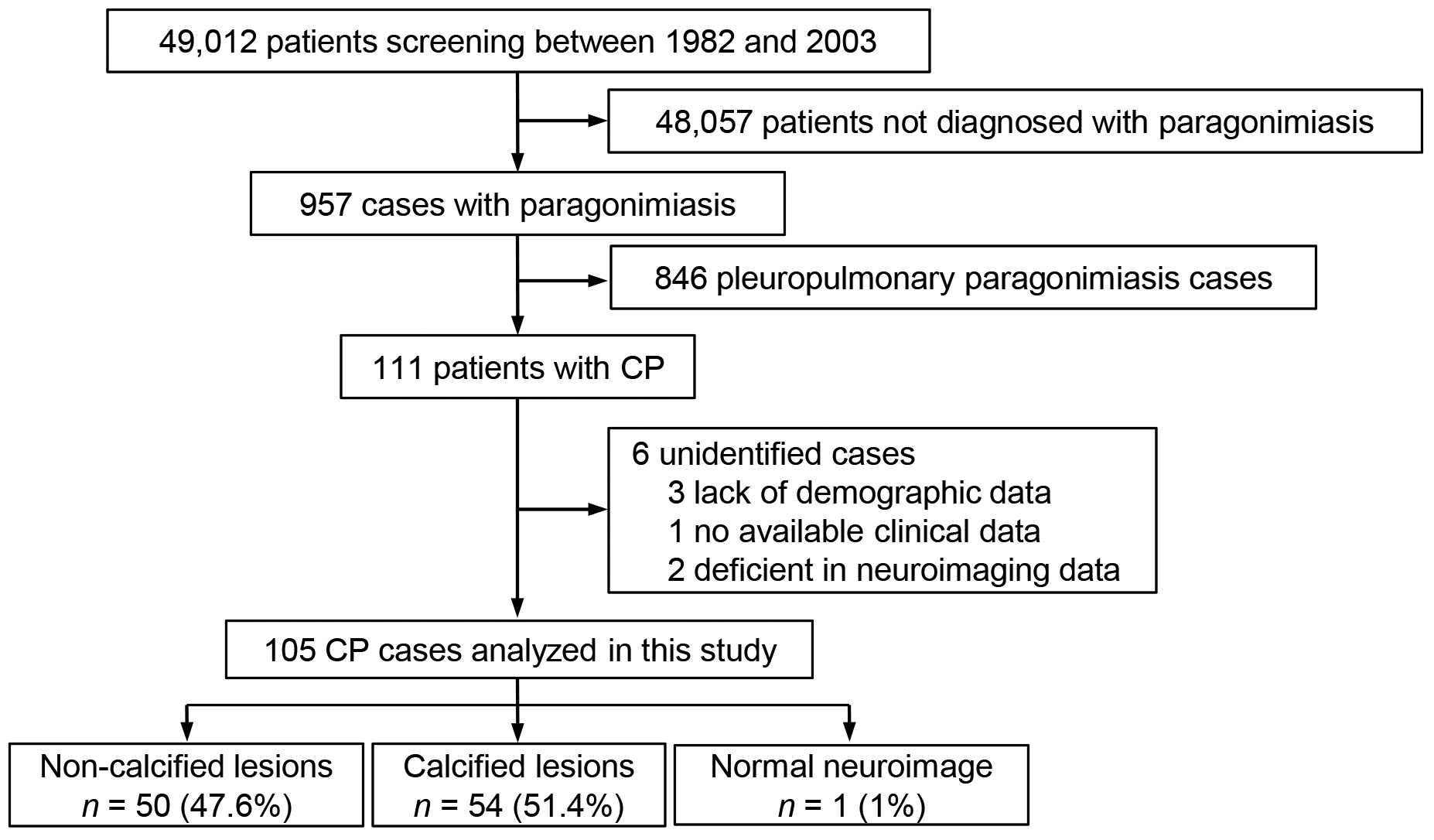

Supplement: S1 Fig — CP = Cerebral paragonimiasis. (TIF) [file pntd.0010240.s002.tif]

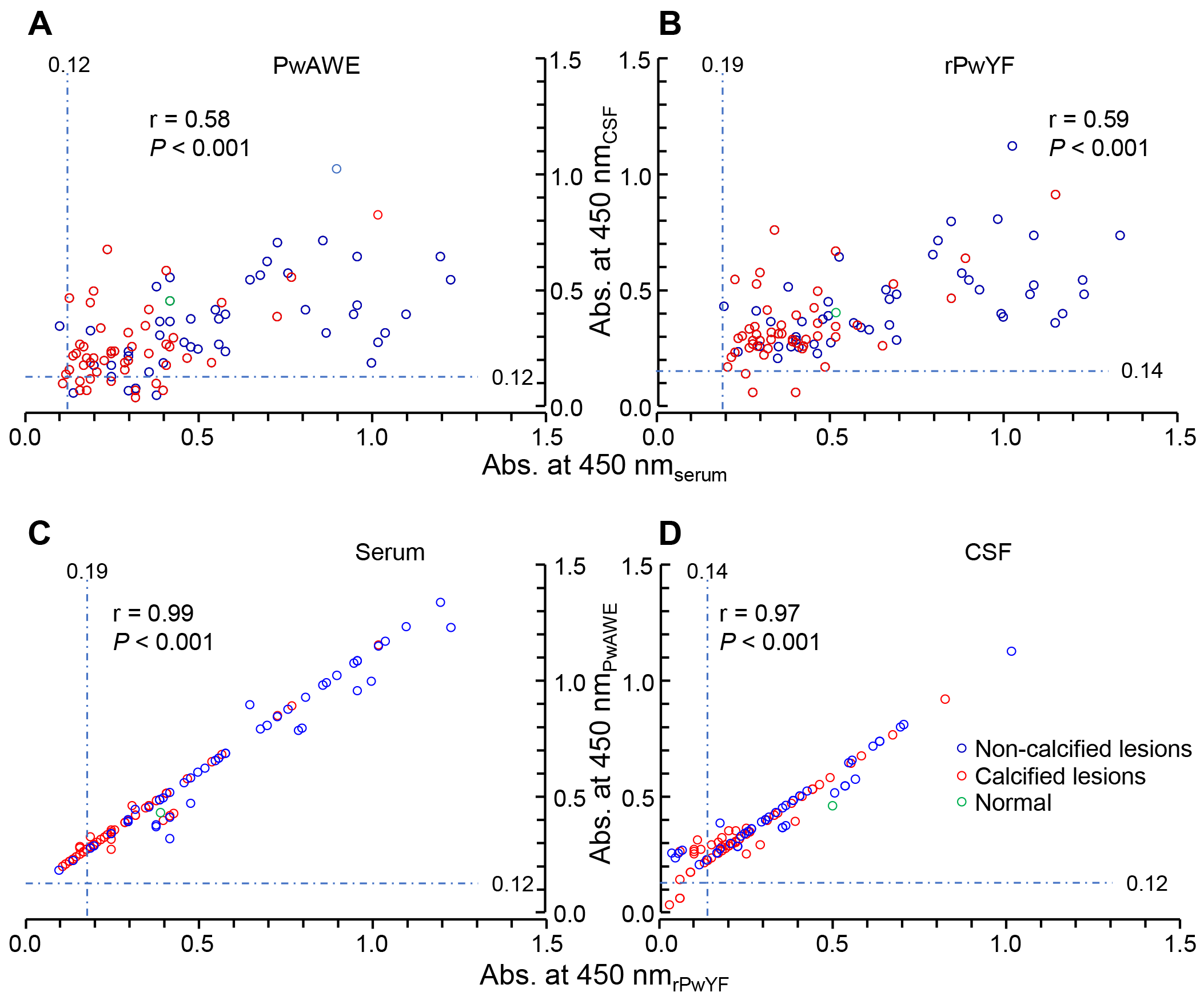

Supplement: S2 Fig — (A and B) Relationship between specific IgG antibody levels against PwAWE and rPwYF in serum and CSF samples from patients with cerebral paragonimiasis. (C and D) Relationship of specific IgG antibody levels between serum and CSF against PwAWE and rPwYF. The horizontal and vertical double dotted blue lines indicate positive criteria. (TIF) [file pntd.0010240.s003.tif]
